# Chromatin remodeling in bone organoids: from epigenetic regulation to disease modeling and therapeutic translation

**DOI:** 10.3389/fgene.2026.1975296

**Published:** 2026-09-28

**Authors:** Yuxin Zheng, Desheng Liu, Jun Hou, Hong Ding, Yingying He

**Affiliations:** 1 School of Clinical Medicine, Qiqihar Medical University, Qiqihar, China; 2 Hospital of Chengdu University of Traditional Chinese Medicine, Chengdu, Sichuan Province, China; 3 School of Clinical Medicine, Chengdu Medical College, The First Affiliated Hospital of Chengdu Medical College, Chengdu, Sichuan, China; 4 Orthopedic Center, Liangjiang Hospital Affiliated to Chongqing Medical University, Chongqing, China; 5 Orthopedic Center, the People’s Hospital of Chongqing Liangjiang New Area, Chongqing, China; 6 Jiulongpo District Maternal and Child Healthcare Hospital, Chongqing, China

**Keywords:** bone disease modeling, bone organoids, chromatin remodeling, epigenetic regulation, skeletal development

## Abstract

Chromatin remodeling contributes to skeletal development, tissue homeostasis, and disease by regulating nucleosome positioning, chromatin accessibility, and histone modifications. Bone organoids can partially reproduce the three-dimensional human bone microenvironment and provide new *in vitro* models for studying skeletal pathophysiology. This review summarizes recent progress in chromatin remodeling and bone organoid research, with a focus on the roles of switch/sucrose non-fermenting (SWI/SNF), imitation switch (ISWI), and chromodomain helicase deoxyribonucleic acid (DNA)-binding (CHD) complexes in bone organoid regulation. We also discuss the relationships between bone organoids and enhancer remodeling, DNA methylation, histone modifications, chromatin organization, and cellular metabolism. In addition, we summarize the applications of emerging technologies, including multiomics, spatial epigenomics, and live cell imaging, in bone organoid studies. The current limitations of these models are also considered. Finally, we discuss potential directions for the further development and refinement of bone organoids. We highlight their potential applications in bone disease research, drug screening, and future clinical translation within the context of chromatin remodeling.

## 1 Introduction

Orthopedic diseases, including osteoporosis, osteoarthritis, osteogenesis imperfecta, and bone tumors, impose a substantial global health burden. This burden is expected to increase with population aging (Compston et al., 2019; Holden et al., 2026). Traditional orthopedic research has relied largely on two-dimensional cell cultures and animal models. However, these systems cannot fully reproduce the cellular diversity and microenvironment of human bone development, homeostasis, and disease (De Pace et al., 2025).Two-dimensional cultures provide limited cell–cell interactions and do not reproduce the three-dimensional organization of the extracellular matrix (ECM). Animal models provide important physiological information, but species-specific differences may affect bone metabolism and drug responses. These differences can limit the translation of some findings to humans. Bone organoids have therefore emerged as complementary human models. They are three-dimensional tissues generated from human pluripotent stem cells (hPSCs), mesenchymal stem cells (MSCs), or other progenitor populations (Hong Y. et al., 2025; Kong et al., 2024). Depending on the culture system, they can reproduce selected features of osteogenic differentiation, matrix deposition, mineralization, and multicellular interactions (Hong Y. et al., 2025). Bone organoids may thus provide a more physiologically relevant platform for studying disease mechanisms and evaluating candidate therapies (Gai et al., 2025; Zhu M. et al., 2026).

Chromatin remodeling is an adenosine triphosphate (ATP)-dependent process that changes nucleosome position, composition, or conformation through ATP hydrolysis (Wang H. et al., 2026). Mammalian ATP-dependent chromatin remodelers are commonly grouped into four families: SWI/SNF, ISWI, CHD, and inositol requiring 80 (INO80)/Swi2/Snf2-Related 1 (SWR1)-related complexes (Monterde and Varela, 2022; Malone and Roberts, 2024). By altering chromatin accessibility, these complexes influence transcription factor binding at cis-regulatory elements. They therefore contribute to cell fate decisions, lineage differentiation, and tissue homeostasis (Koulle et al., 2026; Ponsuksili et al., 2025). In the skeletal system, SWI/SNF complexes, particularly their Brahma-Related Gene 1 (BRG1) and Brahma (BRM) ATPase subunits, participate in the regulation of osteoblasts, chondrocytes, and osteoclasts (Wu W. et al., 2025; Mohd et al., 2025; Zhu S. et al., 2026). Several CHD proteins also regulate skeletal cells, although their functions depend on cell type and developmental stage (Micucci et al., 2015; Sreenivasan et al., 2021). Abnormal activity of these remodelers has been associated with skeletal disorders and metabolic abnormalities (Becker et al., 2025). Defining how chromatin remodeling influences skeletal cell fate may improve our understanding of bone disease and support the development of targeted therapies.

Beyond their use as human skeletal models, bone organoids have also developed into increasingly complex engineered systems. They reproduce selected features of bone development and function rather than the complete architecture of mature bone. Depending on their cellular composition, they may contain mineralized osteogenic matrix, chondrogenic regions, vascular components, or combined bone and cartilage compartments (Wang et al., 2025). Bone organoid research has progressed from simple cell aggregates to engineered systems that incorporate biomaterial scaffolds, microfluidics, bioprinting, and organ-on-a-chip approaches (Hu et al., 2023; Bai et al., 2024). These models can reproduce selected processes, including lineage-specific differentiation, ECM deposition, and mineralization (Duan et al., 2025; Zhang et al., 2021). Compared with conventional *in vitro* models, bone organoids provide a three-dimensional setting that may retain donor-specific genetic features and some epigenetic characteristics. Engineered platforms can also regulate microenvironmental signals in a spatially and temporally controlled manner (Gao et al., 2025). These properties support their use in studies of skeletal development, disease mechanisms, and drug screening.

Although chromatin remodeling is an important regulator of skeletal development, many studies still rely on two-dimensional cultures or genetically modified animals (Wu W. et al., 2025; Cai et al., 2025; Young et al., 2005). However, their ability to capture dynamic chromatin changes within a human skeletal microenvironment remains limited (Ballard et al., 2019; Liu W. et al., 2026). Bone organoids may offer a potentially useful human model for investigating chromatin dynamics in a more physiologically relevant three-dimensional context (Lin et al., 2026). Integrating chromatin remodeling studies with bone organoid technology could improve our understanding of the epigenetic mechanisms underlying bone development and disease. It may also support the development of patient-derived disease models and more individualized therapeutic strategies.

An interdisciplinary framework linking chromatin remodeling with bone organoid research may help clarify the epigenetic regulation of bone development and disease (Figure 1). This review examines the intersection of these fields. It focuses on chromatin mechanisms in bone-forming, cartilage-forming, and bone-resorbing cells, and discusses the potential of patient-derived organoids for disease modeling and therapeutic development.

**FIGURE 1:**
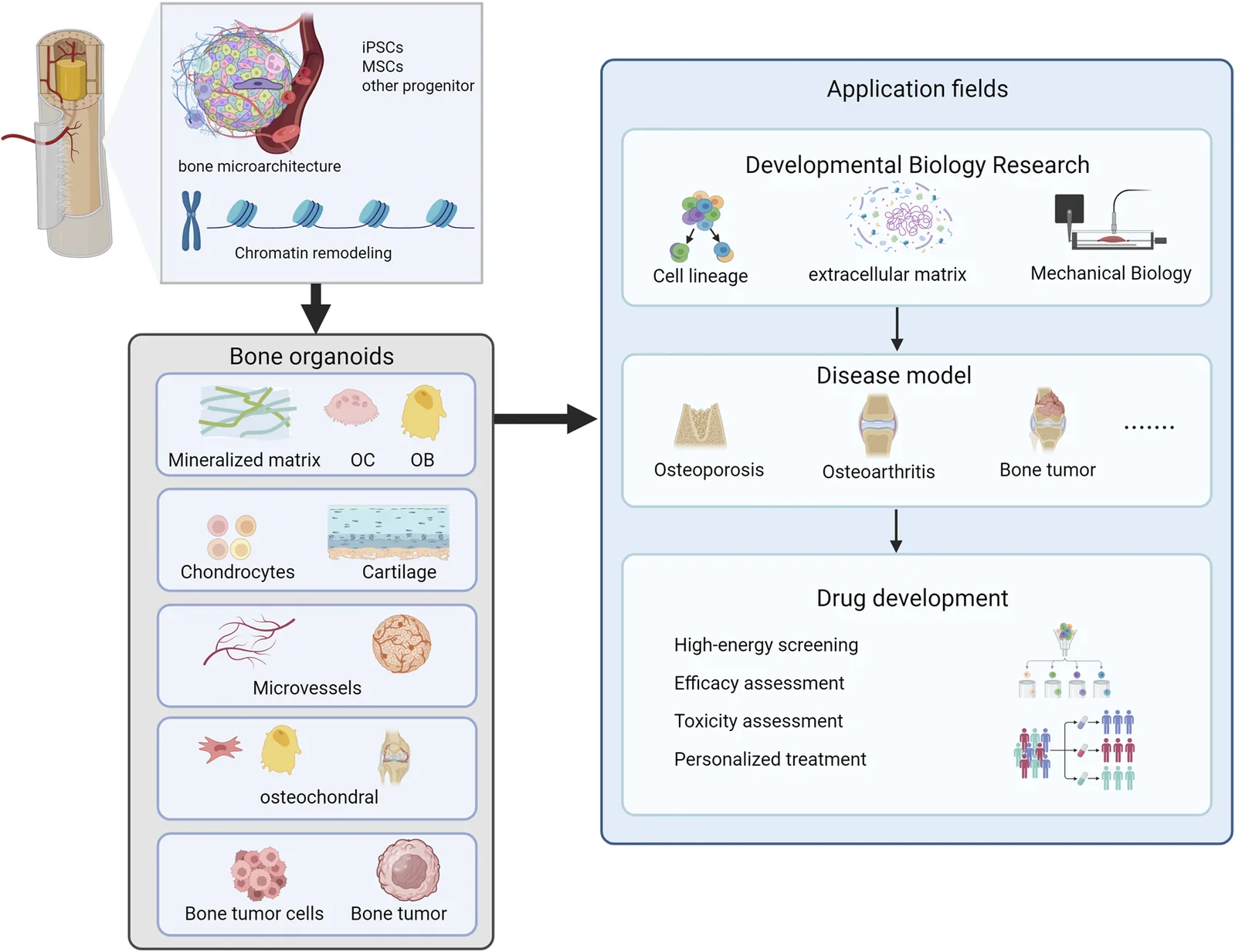
Schematic diagram showing the relationships among the bone microenvironment, bone organoids, and disease-modeling applications. (Created by Biorender).

## 2 Chromatin remodeling

Chromatin remodeling is an important layer of epigenetic regulation. The four major chromatin-remodeling families differ in their catalytic ATPases, associated subunits, and preferred chromatin substrates (Wang H. et al., 2026; Bao and Shen, 2011; Morrison and Shen, 2009; Eustermann et al., 2024; Chen K. et al., 2023). SWI/SNF complexes regulate chromatin accessibility through ATP-dependent changes in nucleosome position and structure (Saha et al., 2024a; Saha et al., 2024b). Their major subfamilies include BRM/BRG1-Associated Factor complex (BAF), Polybromo-Associated BAF complex (PBAF), and Non-Canonical BAF complex (ncBAF) (Chen K. et al., 2023; Lei et al., 2019). Some subunits contain bromodomains that recognize acetylated chromatin. For example, Bromodomain containing 7 (BRD7) is a characteristic component of PBAF, whereas Bromodomain containing 9 (BRD9) is mainly associated with ncBAF (Karim et al., 2020; Jothi et al., 2026; Wang et al., 2022a). These complexes have been implicated in transcriptional regulation and osteoblast differentiation, although their effects depend on the specific subunit and cellular context (Wu W. et al., 2025; Mashtalir et al., 2018). Histone variant exchange is more closely associated with INO80 and SWR1-related complexes. For example, SNF2-Related CREBBP Activator Protein (SRCAP) promotes the deposition of the histone variant H2A.Z (Tan et al., 2026; Yu et al., 2024).

ISWI complexes contribute mainly to nucleosome assembly, spacing, and higher order chromatin organization (Erdel et al., 2010; Eriksson and Clark, 2021). Their SLIDE domains help recognize linker DNA and support the formation of regular nucleosome arrays (Hota et al., 2013). The CHD family contains several functionally distinct subgroups (Malone and Roberts, 2024). Some members possess tandem chromodomains that contribute to the recognition of modified chromatin, whereas others use different targeting mechanisms (Gourisankar et al., 2024). CHD proteins participate in transcriptional regulation, DNA repair, and stem cell maintenance (Liu et al., 2021). INO80/SWR1-related complexes are involved in nucleosome remodeling, histone variant exchange, DNA repair, and replication fork stability (Liu et al., 2021; Clune et al., 2025). SRCAP and the Tat-Interactive Protein 60 (TIP60) complex should be considered distinct chromatin regulatory systems. SRCAP functions mainly as an H2A.Z exchange factor, whereas TIP60 is a K lysine acetyltransferase 5 (KAT5)-containing histone acetyltransferase complex (Tollenaere et al., 2026; Jostes et al., 2024).

Chromatin remodeling does not operate in isolation. It functions within a multilayered epigenetic network that includes DNA methylation, covalent histone modifications, and non-coding ribonucleic acids (ncRNAs) (Shi et al., 2026). These systems interact through epigenetic crosstalk and contribute to coordinated gene regulation. SWI/SNF complexes may influence the methylation state of selected genomic loci through interactions with DNA methyltransferases (DNMTs), methyl-CpG-binding domain (MBD) proteins, and other chromatin-associated factors (Zhu et al., 2013; Banine et al., 2005). Conversely, DNA methylation can affect the recruitment and activity of SWI/SNF complexes, depending on the genomic context and complex composition (Zhu et al., 2013; Wang and Tang, 2023). These reciprocal interactions may contribute to the maintenance of distinct transcriptional states.

Chromatin remodelers also cooperate with histone-modifying enzymes. Active histone marks, such as H3K4me3 and H3K27ac, are often associated with accessible promoters and enhancers, whereas H3K27me3 is linked to Polycomb-mediated repression (Zhou et al., 2026; King et al., 2016). These modifications can influence the recruitment and activity of remodeling complexes, although their effects depend on the remodeler, genomic locus, and cellular context. H3K4me3 is deposited mainly by SET1/complex of Proteins Associated with Set1 (COMPASS) and mixed-lineage Leukemia (MLL)/COMPASS-like complexes, whereas H3K27me3 is catalyzed primarily by Polycomb repressive complex 2 (PRC2) (Guo Y. et al., 2021; Prokopuk et al., 2017). Thus, these marks should therefore not be viewed as universal molecular switches. They are components of context-dependent regulatory networks.

Long non-coding RNAs (lncRNAs) may regulate chromatin remodeling by serving as scaffolds or guides at selected genomic loci (Adams et al., 2017; Mattick et al., 2023). MicroRNAs and circular RNAs can affect the expression of remodeling factors through post-transcriptional mechanisms (Shao et al., 2021; Chen et al., 2021). Their effects on chromatin accessibility are usually indirect.

Three-dimensional chromatin organization provides an additional layer of gene regulation. Topologically associating domains (TADs) are genomic regions with preferential internal contacts (Szabo et al., 2019; Ling et al., 2022; Li KY. et al., 2025). Their boundaries are frequently associated with CCCTC-binding factor (CTCF) and cohesin, although other factors also contribute to their formation and maintenance(Kang et al., 2020). Selected chromatin remodeling complexes may also interact with transcriptional condensates through multivalent protein and chromatin interactions (Malone and Roberts, 2024; Demmerle et al., 2023).

Most of the current evidence concerning these chromatin regulators in skeletal biology has been obtained from bone marrow mesenchymal stem cells, osteoblasts, chondrocytes, osteoclasts, or animal models. These findings provide a useful mechanistic basis for understanding chromatin regulation during skeletal development, but they do not necessarily reflect the behavior of these regulators in bone organoids. Their roles in organoid formation, maturation, and multicellular organization therefore require direct validation in organoid-based systems.

## 3 The molecular mechanisms by which chromatin remodeling regulates bone development

### 3.1 The chromatin regulatory network of osteogenic differentiation

Osteoblast differentiation involves the coordinated and stage-dependent activation of lineage-associated transcription factors, including Runt-related transcription factor 2 (RUNX2), Osterix (SP7), Distal-less homeobox 5 (DLX5), and Activating transcription factor 4 (ATF4) (Kang and Kim, 2026; Chan et al., 2021). The accessibility of their cis-regulatory elements is influenced by ATP-dependent chromatin remodeling, histone modifications, transcription factor binding, and extracellular signals (Chan et al., 2021; Friman et al., 2019).

RUNX2 is a key regulator of osteoblast differentiation and cooperates with SWI/SNF complexes to regulate skeletal gene expression (Takahata et al., 2026). The ATPase subunits BRG1 (*SMARCA4*) and BRM (*SMARCA2*) can alter nucleosome organization and chromatin accessibility. These changes may facilitate the recruitment or activity of osteogenic transcriptional regulators (Chen et al., 2024a). However, SWI/SNF complexes do not directly deposit H3K4me3 or H3K27ac. They may instead cooperate with histone-modifying enzymes and transcription factors to establish active regulatory states at osteogenic promoters and enhancers (Chen K. et al., 2023; Busby et al., 2021). During the early transition of MSCs toward the osteoblast lineage, BRG1-containing complexes may cooperate with RUNX2 and other transcriptional regulators at osteogenic enhancers (Zhu et al., 2024). This interaction may contribute to increased chromatin accessibility at loci involved in osteoblast commitment. BAF45A-containing PBAF complexes have also been implicated in the regulation of chromatin accessibility and gene expression during osteoblast maturation and mineralization (Busby et al., 2021). Whether BRM directly reshapes the SP7 and DLX5 loci during matrix maturation, and whether this mechanism regulates late osteogenic genes such as Bone gamma-carboxylutamate protein (*BGLAP*, Osteocalcin), Integrin-binding sialoprotein (*IBSP*), and Secreted phosphoprotein 1 (*SPP1*), requires further investigation (Elbaz et al., 2024; Wu L. et al., 2025; Fujita et al., 2025). To date, the effects of BRG1 and BRM on osteogenic chromatin remodeling have been characterized primarily in conventional cell models rather than in fully developed bone organoids.

The enhancer landscape is extensively reorganized during osteogenic differentiation. Poised or primed enhancers marked by H3K4me1 and low or absent H3K27ac may acquire H3K27ac in response to lineage-specific signals. They may then become transcriptionally active. This transition is not uniform across all osteogenic enhancers and depends on cell type and differentiation stage (Barral and Dejardin, 2023; Creyghton et al., 2010). ATP-dependent remodelers, including SWI/SNF complexes, may facilitate these changes by repositioning nucleosomes and cooperating with lineage-specific transcription factors (Alver et al., 2017). Enhancer-promoter contacts also contribute to osteogenic gene regulation. During commitment to the osteoblast lineage, lineage-specific chromatin loops and associated epigenomic features undergo dynamic changes (Hao et al., 2022). Altered three-dimensional genome organization may affect skeletal regulatory loci and contribute to disease susceptibility, although the underlying mechanisms remain incompletely defined (Chen XF. et al., 2024). CTCF and cohesin also contribute to genome organization. The orientation of CTCF-binding motifs can influence loop formation through loop extrusion. DNA methylation may affect CTCF occupancy at selected sites, but its impact on osteogenic chromatin loops appears to be context dependent (Hao et al., 2022; Roseman et al., 2025).

### 3.2 Epigenetic regulation of chondrocyte development and endochondral ossification

Chondrocyte specification is coordinated by the SRY-box transcription factor 9 (SOX9) regulatory network and by changes in the chromatin state of cartilage-specific regulatory elements. Recent chromatin profiling has identified SOX9-bound enhancers that contribute to chondrocyte identity and skeletal development (Ichiyama-Kobayashi et al., 2024). During hypertrophic differentiation, SOX9 activity declines, whereas enhancer usage and chromatin accessibility are remodeled (Tan et al., 2018). Chromodomain helicase DNA-binding protein 7 (CHD7) can recognize H3K4me1-associated regulatory regions and has been implicated in enhancer regulation during skeletal development. However, its role in maintaining *Sox9* enhancer accessibility and its functional relationship with Sox9 may vary across developmental contexts (Schnetz et al., 2009; Bajpai et al., 2010). Studies in conventional chondrogenic models suggest that CHD7 may cooperate with SOX9-associated regulatory programs during chondrocyte differentiation. Whether this relationship is maintained in bone organoids remains to be tested.

Hypertrophic differentiation is also associated with changes in nucleosome organization, histone variant dynamics, and three-dimensional chromatin contacts. ATP-dependent remodelers may cooperate with hypertrophy-related transcription factors to regulate genes involved in matrix maturation and mineralization. However, the direct contributions of INO80, SRCAP, and SWI/SNF complexes to these events in hypertrophic chondrocytes remain incompletely defined. Dynamic changes in enhancer-promoter contacts have been reported during chondrocyte development and may contribute to the activation of hypertrophy- and ossification-related genes (Kodama et al., 2022; Long et al., 2022; Collins et al., 2024). These epigenetic changes may support cartilage matrix maturation, mineralization, and vascular invasion during endochondral ossification.

Epigenetic alterations are also associated with chondrocyte senescence and osteoarthritis. Age-related changes in DNA methylation have been observed in articular chondrocytes and may reflect both cellular aging and disease progression (Horvath, 2013). Histone modifications, chromatin accessibility, and inflammatory gene regulation are also altered in osteoarthritic cartilage. However, the formation of canonical senescence-associated heterochromatin foci and the specific involvement of H3K9me3 or H3K27me3 remain context dependent (Liu Z. et al., 2020). These chromatin changes may contribute to the expression of senescence-associated secretory phenotype (SASP)-related inflammatory and catabolic genes, but their causal roles require further investigation.

### 3.3 Chromatin mechanisms of Osteoclast differentiation

Osteoclasts primarily arise from monocyte and macrophage lineage precursors (Wei et al., 2026). Their differentiation is driven by coordinated macrophage colony-stimulating factor (M-CSF) and receptor activator of nuclear factor-κB ligand (RANKL) signaling. M-CSF supports precursor survival and proliferation, whereas RANKL activates Nuclear factor kappa B (NF-κB), Activator protein 1 (AP-1), and Nuclear factor of activated T-cells, cytoplasmic 1 (NFATc1). These factors establish the transcriptional program required for osteoclastogenesis (Cui et al., 2025; Lu et al., 2026). RANKL stimulation is also associated with increased chromatin accessibility at osteoclastogenic enhancers and super-enhancers.

RANKL induces nuclear entry of p65 (RelA) and promotes the activation of osteoclastogenic genes, including Nuclear factor of activated T-cells, *Nfatc1*, Acid phosphatase 5, tartrate resistant (*Acp5, TRAP*), and Cathepsin K (*Ctsk*). The contribution of BRG1 and H3K27ac to this process may depend on the genomic locus and cellular context (Zhu S. et al., 2026; Bae et al., 2023). Microphthalmia-associated transcription factor (MITF) also participates in osteoclast differentiation and bone resorption. Its activity is coordinated with *Nfatc1*, Cellular FBJ murine osteosarcoma viral oncogene homolog (c-FOS), Purine-rich box 1 (PU.1, *SPI1*), and other AP-1 factors (Wu W. et al., 2025). However, evidence for a direct MITF–INO80 mechanism involving H2A.Z deposition at osteoclastogenic enhancers remains limited.

Cell fusion and multinucleation are essential steps in osteoclast maturation. These processes require the coordinated expression of fusion-related genes, including *DCSTAMP* and *OCSTAMP*, together with extensive transcriptional reprogramming (Boyle et al., 2003). Chromatin remodeling complexes may contribute to the accessibility of these loci, but their specific roles in osteoclast fusion remain incompletely defined. In mature osteoclasts, lysosomal enzymes and cytoskeletal proteins support ruffled border formation and bone resorption (Zhu S. et al., 2026). Their expression is regulated by transcription factor activity, chromatin accessibility, and cell signaling. It cannot yet be attributed to a single ISWI or SWI/SNF complex.

Metabolic changes may further influence the osteoclast epigenome. Acetyl-CoA provides acetyl groups for histone acetylation, including the formation of H3K27ac. α-Ketoglutarate (α-KG) acts as a cofactor for ten-eleven translocation (TET) DNA dioxygenases and Jumonji C (JmjC) domain-containing histone demethylases (Bae et al., 2023; Deng et al., 2024; Yang Y. et al., 2026). These metabolites may therefore connect cellular metabolism with the regulation of osteoclastogenic genes. In particular, Jumonji domain containing 3 (JMJD3)-mediated removal of the repressive mark H3K27me3 at the NFATc1 locus has been implicated in RANKL-induced osteoclast differentiation (Stegen et al., 2024). However, the effects of acetyl-CoA and α-KG on DNA methylation, H3K27ac, and H3K27me3 are likely to depend on metabolic state and cellular context (Yang Y. et al., 2026). Direct evidence linking acetyl-CoA metabolism to BRG1 activation at specific osteoclastogenic loci remains insufficient. It is worth mentioning that e vidence for the roles of TET proteins and JMJD3 in osteoclast differentiation has been obtained mainly from osteoclast cultures and related conventional models. These findings may help identify candidate epigenetic regulators in bone organoids containing osteoclast-like cells, but their functions in a multicellular organoid environment remain largely unexplored.

### 3.4 Chromatin basis of intercellular communication in the bone microenvironment

Bone remodeling is coordinated not only by soluble factors such as RANKL and osteoprotegerin (OPG), but also by cell contact, extracellular vesicles, and cell state-dependent chromatin regulation.

Membrane-bound RANKL from osteoblast-lineage cells and osteocytes promotes osteoclast differentiation through RANK. RANKL-containing extracellular vesicles (exosomes) may also stimulate osteoclastogenesis, although their cellular origin and contribution to physiological bone remodeling remain context dependent (Bae et al., 2023; Holliday et al., 2021). Conversely, osteoclastic bone resorption releases matrix-derived factors, including transforming growth factor beta (TGF-β) and insulin-like growth factor 1 (IGF-1). These factors can influence stromal cell recruitment and osteogenic responses (Zhu et al., 2024; Trivedi et al., 2021; Siddiqui and Partridge, 2016). Whether they directly modify SWI/SNF activity in osteoblast-lineage cells remains to be determined.

Bone marrow mesenchymal stem/stromal cells (BMSCs) heterogeneity is partly associated with differences in chromatin accessibility. Studies using single-cell assay for transposase-accessible chromatin sequencing (scATAC-seq) suggest that lineage priming toward osteogenic, adipogenic, or chondrogenic fates is associated with increased accessibility at regulatory regions linked to factors such as RUNX2, Peroxisome proliferator-activated receptor gamma (*PPARG*), and *SOX9* (Walewska et al., 2023). CHD family proteins may contribute to chromatin organization and the control of premature lineage commitment. However, their functions are member-specific and should not be considered a universal barrier to BMSCs multipotency (Shah et al., 2025; Munakata et al., 2025).

Bone microvascular endothelial cells (BMECs) regulate skeletal formation through angiogenic and angiocrine signals (Li S. et al., 2025; Ribatti and d'Amati, 2023). Hypoxia-inducible factor 1 alpha (HIF-1α), vascular endothelial growth factor (VEGF), and Notch pathways contribute to the coordination of vascular growth and osteogenesis. HIF-1α may regulate chromatin accessibility at pro-angiogenic loci, including Vascular endothelial growth factor A (*VEGFA*) and angiopoietin 1 (*ANGPT1*), but direct evidence for the redistribution of specific chromatin remodeling complexes in BMECs remains limited (Carmichael et al., 2025; You et al., 2023). Endothelial Notch ligands can affect neighboring osteolineage cells through juxtacrine signaling. These interactions may influence chromatin accessibility and osteogenic gene expression, although a direct feed-forward mechanism involving SWI/SNF has not been fully established (Perepletchikova et al., 2025; Fernandes et al., 2026). Vascularized bone organoids may therefore help determine whether endothelial signals are sufficient to reproduce the chromatin states observed during skeletal remodeling (Wu W. et al., 2025; Bieluszewski et al., 2023).

## 4 Establishment and validation of bone organoids for chromatin studies

### 4.1 Diversification of bone organoid culture systems

Bone organoid protocols can be broadly classified according to both cell source and differentiation strategy. Common approaches include directed differentiation of human embryonic stem cells (hESCs) or induced pluripotent stem cells (iPSCs), as well as self-organization-based methods (Lou et al., 2025a; Hojo et al., 2024). Directed differentiation protocols leverage the sequential orchestration of small-molecule combinations—principally the Glycogen synthase kinase 3 (GSK3) inhibitor CHIR99021, which activates Wnt/β-catenin signaling, bone morphogenetic protein (BMP) signaling agonists (BMP2 or BMP4), and fibroblast growth factor 2 (FGF2) (Gadomski et al., 2024). This programmatic signaling guides pluripotent stem cells to transition systematically through mesoderm, sclerotome, and osteoprogenitor stages, leading to the formation and mineralization of bone-like matrix (Lamande et al., 2023). The advantage of this protocol is its clearly defined differentiation path and high batch-to-batch reproducibility; however, persistent exogenous signaling may limit activation of endogenous gene regulatory networks during cell self-assembly. In contrast, self-assembly paradigms embed hESCs/iPSCs within matrigel or culture them on low-attachment plates to exploit cells’ intrinsic three-dimensional aggregation capacity and biomechanical cues (Lin et al., 2026; Yin et al., 2025). This approach triggers spontaneous morphogenesis, yielding complex constructs that encompass both chondrogenic templates and bone matrices, and may better capture selected spatial and temporal features of embryonic skeletal development (Kong et al., 2024).

Biomaterial scaffold-assisted engineering strategies optimize the structural stability and functional maturation of bone organoids by mimicking the biochemical and mechanical properties of the native bone ECM (Han et al., 2026; Su et al., 2026). Prior studies have established that composite scaffolds engineered from hydroxyapatite (HA) and collagen potentiate osteoblastic ECM deposition by providing a biomimetic mineralization template (Oh et al., 2025). Concurrently, calcium phosphate ceramic scaffolds, through the precise modulation of their porosity and degradation kinetics, act as microenvironmental instructions that dictate the osteogenic differentiation efficiency of MSCs (Wang W. et al., 2023). Recent evidence underscores that decellularized bone matrix (DBM) scaffolds, by virtue of retaining native collagenous fibrillar alignments and bound growth factor compositions, serve as biologically relevant microenvironments that more potently drive iPSC differentiation into mature osteoblasts while successfully reproducing selected features of the native bone matrix architecture (Domingues et al., 2025; Hossen et al., 2025). The superior performance of DBM/decellularized extracellular matrix (dECM) stems from its dual role: it functions not merely as a passive physical scaffold, but as an active repository that preserves both ultrastructural topographies (e.g., collagen fibrillar alignment) and endogenous matrix-bound growth factors (e.g., BMPs, TGF-β). These biochemical and biophysical cues may influence chromatin accessibility and lineage-specific gene expression during osteogenic differentiation, thereby orchestrating the precise temporal maturation of osteoblasts (Zhou et al., 2022; Shelar et al., 2025).

The size of bone organoids has long remained an unresolved bottleneck. Recent studies indicate that engineering vascularized bone organoids is the key to overcoming the diffusion limit that constrains organoid growth. By establishing three-dimensional co-culture platforms that pair either human umbilical vein endothelial cells (HUVECs) or human induced pluripotent stem cell-derived endothelial cells (iPSC-ECs) with osteolineage cells, researchers can drive the formation of functional and, where demonstrated, perfusable microvascular networks within the supportive biomatrix (Simunovic and Finkenzeller, 2021). Dynamic perfusion culture facilitated by microfluidic technology overcomes mass transport limitations by ensuring a continuous influx of nutrients and oxygen through interconnected microchannels (Sitte et al., 2024). Crucially, the accompanying fluid shear stress acts as a mechanical stimulus that significantly mitigates core hypoxia, enhances interstitial cell viability, and promotes programmatic, uniform matrix mineralization throughout the engineered bone organoids (Han et al., 2026). Furthermore, osteoimmune organoid co-culture systems, by incorporating macrophages or osteoclast precursors, recapitulate the intricate immune-osteoblast crosstalk during coupled bone resorption and formation, thereby providing a pathophysiologically relevant platform to interrogate microenvironment-driven chromatin remodeling under inflammatory stresses (Lou et al., 2025a; Borciani et al., 2020).

### 4.2 Epigenetic and chromatin-level validation of bone organoids

Because culture conditions can affect both cellular composition and epigenetic state, bone organoids should be evaluated at the molecular as well as the structural level. Verifying that bone organoids faithfully recapitulate the epigenetic features of *in vivo* bone tissue is a prerequisite for establishing them as a platform for chromatin remodeling research. Rigorous evaluation of the chromatin accessibility landscape principally relies on two widely used approaches: assay for transposase-accessible chromatin sequencing (ATAC-seq) and DNase I hypersensitive site sequencing (DNase-seq).

Chromatin accessibility analysis reveals that iPSC-derived bone organoids closely mirror primary hMSCs and fetal bone tissues regarding the motif enrichment of open chromatin regions (OCRs) (Tani et al., 2023). This structural fidelity is highlighted by the overlapping topologies of DNase I hypersensitive sites (DHSs) governing the loci of key osteogenesis-driving transcription factors, including RUNX2, SP7, and DLX5 (Hojo et al., 2022; Hojo, 2023). Systematic comparison of global histone modification profiles underscores that the enrichment patterns of the active transcription mark H3K4me3 and the active enhancer mark H3K27ac at osteogenic promoters in osteolineage cells exhibit a stringent positive correlation with those in primary bone tissues (Yi et al., 2019). Concurrently, the repressive deposition architecture of H3K27me3 across alternative adipogenic and chondrogenic lineage-specific loci remains highly conserved, effectively safeguarding appropriate lineage restriction (Li K. et al., 2021).

Notably, the early developmental phase of the bone cell lineage (<4 weeks) is characterized by an elevated baseline of H3K27me3 enrichment, reflecting a regulatory latency wherein the epigenetic barrier enforced by PRC2 has yet to be fully dismantled (Yi et al., 2019). Over prolonged cultivation, concomitant with the progressive deposition of mineralized matrix, this repressive landscape is systematically erased, allowing the global histone modification profile to gradually converge toward the hallmarks of mature bone tissue (Han et al., 2026).

Interrogating the architectural similarity of three-dimensional genome structures fundamentally relies upon high-throughput chromosome conformation capture (Hi-C) and Micro-C technologies (Hao et al., 2022; Liu et al., 2024). Micro-C replaces restriction enzyme digestion with micrococcal nuclease to generate 150–200 bp nucleosomal fragments, successfully resolving both macro-scale topologies and fine-scale chromatin features invisible to conventional Hi-C (Lee et al., 2022). However, the historic requirement of millions of input cells has restricted both assays in rare skeletal populations and miniaturized bone organoid cultures (Metelova et al., 2025). Recent low-input Micro-C protocols reduce the number of cells required for three-dimensional genome analysis, enabling high-quality data generation from as few as 100,000 cells and extending single-nucleosome three-dimensional chromatin mapping to precious, sample-limited osteo-lineage models (Shan et al., 2026).

scATAC-seq profiling of epigenetic heterogeneity uncovers distinct chromatin-state subpopulations within bone organoids, capturing a spectrum of quiescent, proliferative, and terminally differentiated osteoblast subsets (Buenrostro et al., 2015). These subpopulations display pronounced gradients in chromatin accessibility across key osteogenic loci (Chen et al., 2024b), mirroring the cellular architecture observed in native bone remodeling units (Zhu et al., 2024). These organoid-derived epigenetic landscapes offer a robust experimental platform to dissect the stochastic *versus* deterministic regulatory modes of chromatin remodeling that drive cell fate determination (Wu W. et al., 2025).

### 4.3 Live-cell approaches for monitoring chromatin dynamics in bone organoids

Live-cell imaging combined with fluorescent labeling systems provides a powerful tool for the indirect investigation of dynamic higher-order chromatin reorganization. For example, expression of a histone H2B–green fluorescent protein (H2B-GFP) fusion enables global labeling of chromatin and reflects changes in its spatial distribution and local density (May et al., 2023). By utilizing high-resolution confocal microscopy or, more optimally, light-sheet fluorescence microscopy (LSFM) designed for long-term live-cell imaging, researchers can track large-scale chromatin condensation and decondensation events, alongside the spatial mobility of distinct chromatin domains, in real time during bone organoid development. These macroscopic dynamics indirectly reflect underlying epigenetic regulatory mechanisms, including nucleosome remodeling pathways such as sliding and eviction (Liu M. et al., 2025; Gonzalez et al., 2026). However, directly tracking nanoscale events like single-nucleosome sliding within living cells remains highly challenging (Hibino et al., 2024). Although live-cell super-resolution microscopy, including stimulated emission depletion microscopy (STED) and photoactivated localization microscopy (PALM), can map the spatial organization of chromatin and the dynamics of chromatin-associated proteins *in situ* (Hibino et al., 2024; Nho and Kim, 2025), elucidating the precise mechanics of nucleosome sliding still relies largely on *in vitro* single-molecule manipulation and spectroscopic approaches, such as optical or magnetic tweezers and single-molecule Förster resonance energy transfer (smFRET), using reconstituted chromatin substrates (Chen P. et al., 2024).

Monitoring DNA methylation dynamics requires technology selection tailored to the biological question. Global 5-mC levels can be assessed by Enzyme-linked immunosorbent assay (ELISA)-based assays such as MethylFlash, whereas real-time visualization of locus-specific 5-mC changes in living cells necessitates genetically encoded sensors—for example, bimolecular anchor-detector (BiAD) systems or dCas9–MBD fusions that enable direct readout of methylation status at endogenous genomic sites (Kohler et al., 2024; Liu K. et al., 2022). These tools permit tracking of 5-mC deposition and erasure, providing a powerful experimental framework for investigating the temporal activation of TET family dioxygenases during osteogenic differentiation (Chen et al., 2026).

### 4.4 Single-cell and spatial multi-omics in bone organoids

Single-cell scRNA-seq, scATAC-seq, and single-cell cleavage under targets and tagmentation (scCUT&Tag) can be integrated, either through physical co-assay of paired modalities or computational alignment, to construct unified maps linking gene expression, chromatin states, and transcription factor activity (Xiong et al., 2024; Isik et al., 2026). In bone organoid research, integrated scRNA-seq and scATAC-seq profiling resolves temporal chromatin accessibility dynamics that precede gene expression, highlighting pioneer factor-mediated remodeling (Lin et al., 2025; Younesi et al., 2026). Concurrently, scCUT&Tag profiling of histone modifications (e.g., H3K27ac and H3K4me3) identifies subpopulation-specific active regulatory regions and helps nominate potential target genes (Wu et al., 2026). Computational frameworks such as Seurat, Signac, and ArchR effectively integrate these multimodal datasets to reconstruct the temporal cascade of chromatin remodeling along osteogenic differentiation trajectories (Chen D. et al., 2023; Stuart et al., 2021; Granja et al., 2021).

Spatial epigenomic technologies overcome the loss of spatial architecture inherent to conventional single-cell dissociation protocols (Zhang et al., 2023). Specifically, spatial ATAC-seq maps localized chromatin accessibility while preserving precise tissue coordinates, achieved either through *in situ* Tn5 transposition coupled with microfluidic barcoding (Liu Y. et al., 2020) or *via* solid-phase capture on barcoded oligonucleotide arrays (Ll et al., 2023; Deng et al., 2022a). Within bone organoid models, this micro-zonal resolution enables the clear delineation of epigenetic boundaries defining osteogenic *versus* chondrogenic zones. Furthermore, spatial CUT&Tag or epigenetic co-profiling resolves subpopulation-specific enhancer landscapes by mapping active histone modifications, such as H3K27ac and H3K4me3, at near-single-cell resolution (Deng et al., 2022b; Matsushita et al., 2023). To bridge these modalities, emerging computational workflows extend frameworks like Seurat and ArchR to achieve spatial multimodal integration by cross-referencing dissociated scRNA-seq/scATAC-seq coupled datasets with these spatial backbones (Hao et al., 2024; Zhang et al., 2026). Through spatial deconvolution and anchor-based transfer learning, these integrative pipelines successfully project high-resolution multimodal gene regulatory networks directly onto the organoid’s physical coordinates, mapping cell-type-specific regulatory elements *in situ* and, when integrated with chromatin conformation data, inferring candidate enhancer-promoter interactions (Zhang et al., 2026; Li Y. et al., 2025). These spatially resolved technologies are uniquely critical for dissecting how chromatin remodeling operates across the spatially heterogeneous bone microenvironment.

## 5 Current landscape of chromatin remodeling in bone organoid research

### 5.1 Functional roles of chromatin remodeling complexes in bone organoid formation

During bone organoid development, SWI/SNF subunits may show distinct expression patterns at different stages of osteogenic differentiation (Wu W. et al., 2025). Chromatin remodeling complexes regulate cell fate by altering nucleosome position and chromatin accessibility. However, most evidence for these mechanisms comes from osteogenic cell cultures or animal models rather than bone organoids. Along the osteogenic differentiation pathway, BRG1 and BRM may have distinct functions. BRG1 has been linked to early osteoprogenitor commitment and the regulation of osteogenic loci, including *Sp7* and *Dlx5* (Young et al., 2005; Rashid et al., 2014). It may promote chromatin accessibility at these loci and is associated with active marks such as H3K4me3 and H3K27ac. However, BRG1 should not be described as directly depositing these histone marks. BRM has also been implicated in later stages of osteoblast differentiation and matrix mineralization, including the regulation of genes such as *Bglap* and *Ibsp* (Ma et al., 2022). The relative contributions of BRG1 and BRM may change during differentiation, but a defined BRG1-to-BRM switch has not been established in bone organoids.

In osteoclasts, AT-rich interaction domain 1A (ARID1A) contributes to the regulation of cell fate during the transition from proliferation to differentiation (Du et al., 2024). It may cooperate with transcription factors and chromatin-associated proteins at osteoclastogenic regulatory elements. However, direct evidence for ARID1A-containing condensates with BRD4 and PU.1 at the NFATc1 super-enhancer remains limited. The functional balance between cBAF and ncBAF may also influence osteoclast differentiation, although this relationship requires further study in bone organoid models (Wu L. et al., 2025). This suggests that the efficiency of osteoclast differentiation in bone organoids may be exquisitely sensitive to the relative abundance of these SWI/SNF subunits.

Metabolic signals may influence chromatin during osteoclast activation. Glycolytic and mitochondrial metabolism can increase the availability of citrate and acetyl-CoA, which are used for histone acetylation (Yang Q. et al., 2026; Guo Q. et al., 2021). These changes may affect H3K27ac at osteoclastogenic enhancers (Bae et al., 2023). Although the BRG1 bromodomain can recognize acetylated chromatin, this does not by itself demonstrate that acetyl-CoA directly activates BRG1 (Zhang et al., 2024). The extent to which nutrient availability affects osteoclast differentiation through this pathway remains to be determined in bone organoids.

Chromatin remodeling may also cooperate with DNA demethylation during osteogenic differentiation. TET enzymes oxidize 5-mC and may facilitate the activation of osteogenic loci, including *Sp7* (Montecino et al., 2020; Sepulveda et al., 2017). Loss of this epigenetic synergy in bone organoid culture can stall osteogenic differentiation or skew lineage output (Dusadeemeelap et al., 2022). Overall, chromatin remodeling appears to regulate skeletal cell differentiation in a stage- and lineage-dependent manner. Future studies should determine whether these mechanisms are reproduced in organoids with defined cellular composition and maturation state.

It is important to distinguish between direct evidence from bone organoids and evidence extrapolated from conventional skeletal models. At present, many mechanistic studies of BRG1, BRM, ARID1A, CHD7, TET proteins, and JMJD3 have been performed in bone marrow mesenchymal stem cells, osteoblasts, chondrocytes, osteoclasts, or animal models. These studies provide candidate mechanisms and experimental targets for bone organoid research, but they do not establish that the same regulatory relationships operate in organoids.

### 5.2 Organoid models of chromatin remodeling defects in bone diseases

Bone organoids are primarily used to investigate local cellular and multicellular mechanisms, including cell differentiation, chromatin regulation, matrix formation, and interactions between osteogenic and osteoclastic lineages. They should not be considered complete models of systemic bone metabolic diseases. Systemic conditions such as osteoporosis involve endocrine signals, circulating factors, immune regulation, vascular changes, and mechanical inputs that are not fully reproduced in current organoid systems. Therefore, findings obtained from bone organoids should be interpreted as mechanisms operating within a defined local microenvironment rather than as a complete representation of whole-body bone metabolism.

Bone organoids provide a controlled *in vitro* platform for dissecting the causal links between chromatin remodeling abnormalities and skeletal pathology (Kong et al., 2024). In osteoarthritis, inflammatory cytokines and abnormal mechanical loading alter gene regulatory programs in chondrocytes. NF-κB, AP-1, and RUNX2 may bind to disease-associated regulatory regions and promote the expression of matrix-degrading enzymes and inflammatory mediators (Kramer et al., 2025; Zhao and Liu, 2026). Because these changes occur mainly in cartilage, osteoarthritis models should preferably use cartilage or osteochondral organoids rather than bone organoids alone. Such models may help evaluate epigenetic drugs that target dysregulated enhancers.

In osteoporosis, altered lineage allocation of MSCs contributes to reduced bone formation (Liu C. et al., 2026). SWI/SNF complexes participate in the regulation of osteogenic and adipogenic gene programs (Nguyen et al., 2015). The relative roles of BRG1 and BRM appear to depend on cell type and differentiation stage. Therefore, their functions should not be reduced to a simple opposition between osteogenesis and adipogenesis. Disruption of these regulatory networks may favor adipogenic differentiation and contribute to bone loss (Wang et al., 2020). Trabecular bone organoids may help model abnormal bone remodeling and evaluate interventions that promote osteogenic differentiation (Bai et al., 2024; Park et al., 2021). However, these models cannot fully reproduce systemic osteoporosis, which is influenced by estrogen deficiency, circulating hormones, immune signals, vascular changes, and mechanical loading. Thus, bone organoids may be useful for studying selected cellular mechanisms associated with osteoporosis, but their results should not be interpreted as a complete model of the systemic disease.

Mechanical cues, including fluid shear stress and compressive loading, influence skeletal cell behavior and may affect chromatin state (Deng and Wan, 2025). Mechanical stimulation can activate calcium-dependent signaling and promote cooperation between BRG1 and RUNX2 at osteogenic loci (Wu W. et al., 2025; Liu et al., 2023). In contrast, simulated microgravity has been associated with impaired osteoblast differentiation and altered histone acetylation in conventional cell models (Tosi et al., 2026; Hu et al., 2022). Whether similar changes occur in bone organoids has not been established. Testing this in a three-dimensional organoid system would provide a much-needed cellular basis for understanding spaceflight-induced bone loss.

Chromatin remodeling abnormalities are also relevant to bone-associated malignancies. In osteosarcoma, reduced BRD7 expression has been associated with poor clinical outcome, whereas changes in BAF155 (*SMARCC1*) may affect tumor-related transcriptional programs (Rafati et al., 2026). In Ewing sarcoma, BRG1 and *ARID1A* may interact with the EWS RNA-binding protein 1 – Friend leukemia integration one transcription factor (EWS-FLI1) fusion protein and contribute to oncogenic gene regulation (Sohn and Libich, 2025; Selvanathan et al., 2019). Synovial sarcoma is primarily a soft-tissue sarcoma, although it can involve bone. It should therefore be discussed as a sarcoma relevant to skeletal disease rather than as a typical bone tumor. Gene-edited organoids may help model selected chromatin remodeling defects, but direct evidence in bone tumor organoids remains limited (Li J. et al., 2021; Mittal and Roberts, 2020).

Genetic syndromes further illustrate the relationship between chromatin remodeling and skeletal development. Coffin-Siris syndrome and Nicolaides-Baraitser syndrome are associated with mutations in ARID1B and *SMARCA2*, respectively. Both can include skeletal abnormalities and delayed development (Pascolini et al., 2020; Mari et al., 2015). These mutations may affect the activity of BAF complexes rather than a single downstream gene (Mittal and Roberts, 2020; Pascolini et al., 2020). Bone organoids derived from patient iPSCs can track how these mutations progressively alter chromatin opening programs during osteogenic differentiation while preserving the individual genetic background.

Many bone organoid models still lack a complete vascular, immune, and metabolic microenvironment. This limitation may reduce their ability to reproduce disease-associated chromatin changes. Other challenges include variable differentiation efficiency, limited gene-editing efficiency, and epigenetic drift during long-term culture. Single-cell and spatial analyses may help define these limitations and improve the disease relevance of organoid models.

### 5.3 Chromatin regulation in bone regeneration and repair

Bone regeneration involves hematoma formation, inflammation, callus formation, and subsequent remodeling by osteoblasts and osteoclasts (Sheen et al., 2026). Chromatin regulation is likely to contribute to the timing of these transitions, including the switch from MSCs proliferation to osteogenic commitment and the onset of matrix mineralization (Iyer, 2025). BRG1 has been implicated in the regulation of early osteogenic genes, including *Runx2* and *Sp7* (Dusadeemeelap et al., 2022). BRM may contribute to later stages of matrix maturation and the expression of mineralization-related genes such as *Ibsp* and *Bglap* (Chavez et al., 2024). These findings suggest that BRG1 and BRM may have temporally distinct functions. However, failure of a defined BRG1-to-BRM transition has not been directly shown to arrest fracture healing or bone organoid maturation.

DNA demethylation is just as critical. TET enzymes oxidize 5-mC and may facilitate the activation of osteogenic loci, including Sp7 (Sepulveda et al., 2017). During fracture healing, the interaction between inflammatory signaling, DNA methylation, and chromatin remodeling may influence MSCs fate (Iyer, 2025).

Three-dimensional bone organoids provide a model for studying selected aspects of bone repair that are difficult to reproduce in monolayer cultures (Huang et al., 2026). They recapitulate the soft-to-hard callus progression, and vascularized variants now allow study of angiogenesis-osteogenesis coupling (Lou et al., 2025b). In this context, bone organoids are most appropriate for studying local events during bone repair, such as osteogenic differentiation, matrix mineralization, vascularization, and interactions between osteoblast-lineage cells and osteoclast-lineage cells. They do not fully reproduce the endocrine, immune, vascular, and mechanical conditions that regulate fracture healing *in vivo*.

Bukhari et al. showed reported effects of estrogen deficiency on vascular patterning and mineralization should be interpreted according to the specific organoid composition and experimental design (Bukhari et al., 2025). Zhu et al. further used dynamic hydrogels to support woven bone organoid formation, achieving robust bone regeneration in calvarial defect models (Zhu M. et al., 2025). Yet most models still lack immune cells and nerve supply, so they miss how inflammatory macrophage polarization shapes MSC chromatin states in the early phase (Lou et al., 2025a). Up to now, Whether single-cell epigenetic heterogeneity in organoids mirrors native bone remodeling units also remains untested. Integrating single-cell and spatial epigenomic approaches may help address this question.

### 5.4 Bone organoids in drug screening and epigenetic therapy

Bone organoids retain the cellular heterogeneity and three-dimensional architecture of native bone (Hong Y. et al., 2025). They mimic drug penetration and metabolism more faithfully than traditional two-dimensional cultures, making them an attractive platform for epigenetic drug screening (Kong et al., 2024; Lou et al., 2025a).

Histone deacetylase (HDAC) inhibitors are among the most studied epigenetic agents. N-(2-Aminophenyl)-4-[N-(pyridin-3-ylmethoxycarbonyl)aminomethyl]benzamide (LMK-235), a selective HDAC4/5 inhibitor, suppresses osteoclast differentiation by downregulating NF-κB and p-Smad2/3 signaling through HDAC4 inhibition (Chen C. et al., 2024). It also promotes osteoblast mineralization by enhancing RUNX2 expression. *In vivo,* LMK-235 alleviates Lipopolysaccharide (LPS)-induced calvarial osteolysis and accelerates bone defect repair. Broad-spectrum HDAC inhibitors such as Trichostatin A (TSA) increase histone acetylation and enhance the osteogenic potential of muscle-derived extracellular vesicles, offering an indirect route to promote bone formation (Chen M. et al., 2024).

In the field of drug screening for bone tumors, Bromodomain and extraterminal domain (BET) inhibitors show promise against bone tumors. JQ1, a thienotriazolodiazepine-based BET domain inhibitor, blocks the vicious cycle between bone tumors and resorption by inhibiting both osteoblast and osteoclast differentiation. PROTAC compounds such as MZ1 and dBET57 have been used to degrade BRD4 and show activity in osteosarcoma models (Lang et al., 2024). Enhancer of zeste homolog 2 (EZH2) inhibitors address myeloma-induced bone destruction. EZH2 silences tumor suppressors such as epithelial membrane protein 1 (EMP1) through H3K27me3 deposition, driving myeloma proliferation and osteoclastogenic cytokine release (Liu R. et al., 2025). EZH2 inhibition significantly reduces osteolytic lesions in animal models. These inhibitors may also upregulate CD38 expression and enhance antibody-dependent cellular cytotoxicity, providing a rationale for combination therapy (Chemlal et al., 2023). Whether these effects can be reproduced in bone organoids containing myeloma cells and osteoclast precursors remains to be tested.

DNA methylation inhibitors also affect skeletal homeostasis. 5-aza-dC reverses aberrant hypermethylation at osteogenic gene promoters in MSCs, restoring Wnt/β-catenin signaling and promoting osteogenic commitment (Adamik et al., 2017; Atienza Parraga et al., 2025). Because 5-aza-dC acts broadly on DNA methylation, its effects should be evaluated together with cell viability, lineage composition, and off-target transcriptional changes.

The main challenge is to establish organoid models that reproduce relevant vascular, immune, and disease-specific features. Such models may improve epigenetic drug screening by linking molecular responses to matrix formation, cellular composition, and tissue organization. Single-cell analysis could further reveal cell-type-specific drug effects, but its predictive value requires validation against patient samples and clinical outcomes. Notably, bone organoids alone cannot reproduce systemic pharmacokinetics, endocrine regulation, circulating drug concentrations, or drug metabolism by organs such as the liver. Their results should therefore be interpreted as local tissue responses rather than whole-body therapeutic effects.

## 6 Current limitations and challenges in bone organoid research

### 6.1 Technical limitations

Data sparsity in single-cell chromatin analysis is a fundamental bottleneck (Luo et al., 2024). Single-cell Hi-C can measure chromatin contacts in individual cells, but the resulting contact maps are often too sparse to reconstruct fine-scale three-dimensional structures reliably (Noh et al., 2025; Dautle and Chen, 2025). Super-resolution imaging provides high spatial resolution, but it usually covers a limited genomic region and has relatively low throughput (Hong F. et al., 2025). These complementary limitations make it difficult to link three-dimensional chromatin organization with local epigenetic states in the same cell.

Spatial epigenomic methods also involve a trade-off between spatial resolution, signal quality, and sample input (Xie et al., 2024). Spatial ATAC-seq and Spatial CUT&Tag preserve positional information, but their performance can be affected by tissue size, cell number, signal recovery, and sample processing. This is particularly relevant to small and heterogeneous bone organoids, in which limited material and uneven cell distribution may reduce data quality (Hong F. et al., 2025). More fundamentally, no existing method can simultaneously assess metabolism, gene expression, epigenetic modifications, and chromatin remodeling within the same single cell (Geisenberger et al., 2025). This limitation makes it difficult to determine how metabolic changes are coupled to chromatin remodeling during osteogenic differentiation (Wang X. et al., 2026). In addition, most available approaches provide a snapshot rather than a continuous measurement of these processes.

### 6.2 Biological complexity

Bone tissue is not a simple collection of osteoblasts. It contains vascular networks, innervation, immune cells, and mechanosensory systems that continuously shape local chromatin environments through cell-cell crosstalk (Wang et al., 2022b). Most current bone organoids do not yet reproduce a fully functional vascular, neural, or immune microenvironment (Ma et al., 2023; Zhu Z. et al., 2025). The absence of these components limits the study of several biological processes. Limited vascularization can affect oxygen and nutrient transport. The lack of immune cells restricts the analysis of inflammation, macrophage polarization, and osteoclast activation. The absence of neural components also limits the investigation of neuro-bone interactions and their potential effects on osteogenic differentiation (Zhu Z. et al., 2025; Liu J. et al., 2025). Thus, the epigenetic changes observed in current organoids may not fully represent those occurring in native bone tissue.

Mechanical loading is another important limitation. Native bone undergoes constant compression, tension, and shear (Liu P. et al., 2022; Vico et al., 2000). These stimuli activate calcium-dependent and kinase-mediated signaling pathways. They may also influence BRG1-associated chromatin regulation and other factors involved in osteogenic gene expression (Flowers et al., 2009; Flowers et al., 2014). However, most bone organoid systems do not reproduce the magnitude, frequency, and direction of physiological loading. Without appropriate mechanical stimulation, organoid-derived chromatin states may become less representative of the mechanically active bone microenvironment. This may affect osteoblast differentiation, matrix deposition, mineralization, and the coordination between bone formation and resorption. Perfusion systems, mechanical bioreactors, and tunable hydrogels may help address this limitation, although their effects on chromatin remodeling require direct evaluation.

### 6.3 Current defects in bone organoids

Immaturity is the most prominent problem. Most protocols stall at the fetal or early developmental stage and fail to acquire the epigenetic landscape of adult bone (Lou et al., 2025a). This difference may reflect incomplete maturation of cellular composition, extracellular matrix, vascularization, and chromatin state.

Some osteogenic loci, including *BGLAP* and *IBSP*, may retain an immature regulatory configuration. This may involve persistent repressive marks, incomplete acquisition of enhancer-associated H3K27ac, or, in some developmental contexts, the coexistence of H3K4me3 and H3K27me3 (Carrasco et al., 2023; Stetsiv et al., 2025). However, the presence of a bivalent state should be confirmed at individual loci rather than assumed for all mineralization-related genes. Long-term culture introduces additional sources of variability. As organoids increase in size, limited diffusion may contribute to central hypoxia, necrosis, cellular senescence, and reduced functional activity (An et al., 2025; Sun et al., 2023). These changes may be accompanied by alterations in chromatin accessibility and gene expression. Such culture-associated changes can complicate the distinction between developmental maturation and pathological or stress-related epigenetic drift.

Batch-to-batch variation is also influenced by the starting cell population, donor characteristics, culture medium, induction timing, and matrix composition. Matrigel and other extracellular matrix preparations may vary between lots, which can affect organoid morphology, cell composition, and chromatin profiles (Kong et al., 2024; Lin et al., 2026). Standardized cell characterization, defined matrix materials, controlled culture conditions, and common quality-control criteria will be necessary for reliable comparison across laboratories.

Gene editing in bone organoids remains technically challenging and is not yet readily scalable for high-throughput functional screening. Editing efficiency may vary with organoid size, matrix composition, cell heterogeneity, editing method, and developmental stage (Bai et al., 2024). Several strategies may improve editing efficiency, including defined synthetic hydrogels (Giri and Rajesh, 2026), optimized electroporation (Abubakar et al., 2026), microfluidic perfusion (Quintard et al., 2024; Moshksayan et al., 2025), and more rigorous quality control (Torres-Montoya et al., 2026). Nevertheless, these approaches require validation in bone-specific models (Lou et al., 2025a). In our view, the main limitation is not simply poor delivery, but the lack of standardized methods for measuring editing efficiency across different cell populations and organoid regions. Chromatin data from bone organoids should therefore be interpreted cautiously. Editing rate, spatial distribution, cell composition, matrix conditions, and organoid size should be reported alongside chromatin-accessibility and transcriptional data.

Aging-associated chromatin changes remain largely untested in bone organoids, which are mainly used to study osteogenic differentiation, tissue formation, and skeletal disease modeling (Bai et al., 2024). It is therefore unclear whether these models reproduce age-related DNA methylation changes, chromatin reorganization, or senescence-associated heterochromatin foci (SAHF). SAHF has been reported in selected senescence models, but its formation is not a universal feature of cellular senescence (Narita et al., 2003). DNA methylation clocks may help assess biological age, although their application to bone organoids requires tissue-specific validation (Horvath and Raj, 2018; Knight et al., 2024). In addition, MSCs expansion can cause replicative senescence and DNA methylation changes, whereas iPSCs reprogramming may reset several age-associated epigenetic features (Franzen et al., 2021; Tesakov et al., 2026; Kim et al., 2010). The stability of epigenetic memory during serial passaging of bone organoids remains unclear. Long-term culture may cause drift in epigenetic marks inherited from iPSCs or MSCs. Whether this compromises experimental reliability remains to be tested. Future studies should combine age-defined donor cells, passage-controlled cultures, DNA methylation analysis, and chromatin profiling to establish reliable aging models of bone organoids.

## 7 Prospects for future research

### 7.1 Technological innovation

Future advances in bone organoids should move beyond bone formation and aim to reconstruct a functional skeletal microenvironment. Vascularization is a primary requirement. Endothelial cells, pericytes, and angiogenic factors may be incorporated to generate perfusable vascular networks. Microfluidic channels may further improve oxygen and nutrient delivery while modelling vascular–osteogenic signaling (Bai et al., 2024; Gao et al., 2025). Innervation and immune integration are also essential. Sensory neurons, macrophages, and bone-marrow immune cells could help define neuroimmune regulation of bone formation and remodeling. However, their cellular ratios, spatial organization, and long-term stability remain poorly standardized (Hong Y. et al., 2025; Kong et al., 2024). Organ-on-chip platforms can integrate fluid shear stress, oxygen gradients, mechanical loading, and multicellular co-culture. Future designs should improve material biocompatibility and establish real-time imaging and standardized readouts for mineralized tissues (Liu K. et al., 2025).

Live-cell imaging of chromatin dynamics could reveal changes in chromatin compaction, enhancer activity, and transcription-factor occupancy during osteogenic differentiation or mechanical stimulation. Such imaging should be combined with single-cell ATAC-seq, CUT&Tag, and spatial transcriptomics rather than used as a stand-alone endpoint (May et al., 2023; Xia et al., 2026). Artificial intelligence (AI)-based models may integrate multi-omics, imaging, and mechanical data to predict links between chromatin states and mineralization phenotypes. Independent validation will be essential, because prediction does not establish causality (Elfatimi et al., 2025; Ding PA. et al., 2026; Ding P. et al., 2026; Yuan et al., 2025; Ding et al., 2026). CRISPR/Cas systems can be used to modify chromatin remodelers, enhancers, and disease-associated genes. Inducible, reversible, and low-off-target strategies should be prioritized. Single-cell sequencing should also be used to assess clonal heterogeneity and genomic stability (Liu M. et al., 2025; Park and Kim, 2025). Future studies could gradually advance the development of standardized bone organoids containing vascular, neural, and immune components. Reproducible chromatin-dynamic measurements may also be incorporated into platform quality-control systems to improve the physiological relevance of these models and the reliability of the experiments.

### 7.2 Frontiers in mechanistic research

Bone organoids provide a useful model for examining the temporal regulation of skeletal development. Future studies should define the critical windows of osteogenic differentiation, matrix maturation, and mineralization (Liu C. et al., 2026). These stages should be analysed together with single-cell transcriptomics, single-cell chromatin-accessibility profiling, and lineage tracing. Current evidence indicates that SWI/SNF-related complexes and BAF45 family members contribute to BMP–RUNX2-mediated activation of osteogenic genes. However, the timing and cell-state dependence of these remodeling factors remain insufficiently defined (Busby et al., 2021; Xu et al., 2012). Inducible protein-degradation systems, CRISPR interference, and time-resolved multi-omics may help distinguish the causal sequence linking chromatin opening, transcription-factor binding, and gene expression. Biomolecular phase separation may contribute to enhancer–promoter interactions, transcriptional condensate formation, and the spatial organization of chromatin (Luo et al., 2021; Li et al., 2024). The condensation behaviour of osteogenic regulators, including RUNX2 and Yes-associated protein (YAP)/Transcriptional coactivator with PDZ-binding motif (TAZ), could provide a mechanistic link between mechanical stimuli and osteogenic transcription (Chen et al., 2025; Katsianou et al., 2025). However, much of the current evidence is based on condensate-like structures or correlative observations (Niu et al., 2023; Wang X. et al., 2023). Whether phase separation is necessary and specific in bone organoids remains unresolved.

Nutrient-sensing pathways also deserve closer attention. AMP-activated protein kinase (AMPK), mechanistic target of rapamycin complex 1 (mTORC1), and hypoxia-responsive pathways detect changes in energy availability, amino acids, oxygen supply, and cellular metabolism (Guney Kalkan and Ceylan Unlusoy, 2025; Chun and Kim, 2021). They may regulate osteogenic differentiation by altering ATP production, histone modifications, and the activity of chromatin-remodeling enzymes (Zhu et al., 2024). Their effects in bone organoids, however, are likely to depend on cell composition, medium formulation, and oxygen gradients. Integrating metabolic flux, chromatin state, and mineralization readouts may therefore be important. These relationships should also be tested in vascularized organoids and under controlled mechanical stimulation. Overall, time-resolved and multiscale perturbation systems may help clarify the coordinated interactions among metabolism, phase separation, and chromatin remodeling, and may gradually improve the mechanistic value of bone organoids.

### 7.3 Prospects for clinical translation

The clinical value of bone organoids lies partly in their ability to link patient-specific features with functional phenotypes. Patient-derived mesenchymal stem cells or induced pluripotent stem cells may be used to reproduce individual differences in osteogenesis, mineralization, and the bone marrow niche. When combined with genomic, epigenomic, and chromatin-accessibility analyses, these models may help identify disease-associated chromatin-remodelling abnormalities. This approach could support studies of osteoporosis, osteogenesis imperfecta, and other rare skeletal disorders. It may also contribute to patient stratification and treatment selection. However, patient-derived bone organoids are still limited by small sample numbers, batch-to-batch variation, and incomplete maturation. Whether their phenotypes reliably reflect pathological changes in patients remains to be determined.

In regenerative medicine, bone organoids may serve as an intermediate platform for evaluating cells, scaffolds, growth factors, and gene-editing strategies. Their three-dimensional organization and cell–cell interactions are more representative of the bone microenvironment than conventional two-dimensional cultures. Nevertheless, substantial barriers remain before clinical implantation can be considered. Vascularization, innervation, immune regulation, mechanical stability, and long-term integration are likely to be critical determinants of therapeutic feasibility. Chromatin-remodelling states may also affect the stability, differentiation potential, and stress responses of transplanted cells. Therefore, chromatin accessibility, key histone modifications, and cell-fate stability should potentially be incorporated into quality-control procedures before progression to animal studies or clinical testing.

Drug development may be one of the most feasible translational applications of bone organoids. These models could support the screening of osteogenic and antiresorptive drugs, while revealing drug-induced changes in chromatin states and cellular phenotypes. However, their ability to predict clinical efficacy remains uncertain. Tissue maturity, drug exposure, and limited immune components may affect treatment responses. Standardized protocols, multicentre validation, and clinically relevant outcome measures will therefore be needed to establish reliable predictive biomarkers. These steps may gradually support the use of bone organoids in disease modelling and drug evaluation.

## 8 Conclusion

Chromatin remodeling is a key molecular layer in bone organoid development and functional maintenance. By regulating three-dimensional genome architecture and transcriptional accessibility, it helps shape cell-fate decisions and osteogenic programs. Bone organoids provide a distinctive human *in vitro* platform for studying these processes. Recent work has established the feasibility and potential value of combining bone organoids with chromatin biology. However, important technical barriers remain. These include incomplete tissue maturation, restricted cellular diversity, limited vascular and immune components, and considerable batch-to-batch variation. Standardized culture conditions and robust quality-control criteria are therefore still needed.

In the longer term, specific bone organoids may support epigenetic precision medicine for degenerative bone diseases, skeletal developmental disorders, and rare genetic conditions. They may also provide a useful platform for regenerative medicine and mechanism-based drug development. However, their clinical utility will depend on whether organoid phenotypes can be linked reliably to patient outcomes. Further progress may therefore come from integrating patient-derived models, spatial chromatin profiling, vascularized or immune-competent systems, and longitudinal clinical validation.
